# Towards Integrated Multi-Trophic Aquaculture: Lessons from Caprellids (Crustacea: Amphipoda)

**DOI:** 10.1371/journal.pone.0154776

**Published:** 2016-04-28

**Authors:** José Manuel Guerra-García, Ismael Hachero-Cruzado, Pablo González-Romero, Pablo Jiménez-Prada, Christopher Cassell, Macarena Ros

**Affiliations:** 1 Laboratorio de Biología Marina, Departamento Zoología, Facultad de Biología, Universidad de Sevilla, Sevilla, Spain; 2 IFAPA–El Toruño, Camino Tiro Pichón s/n, El Puerto de Santa María, Cádiz, Spain; 3 School of Biological Sciences, University of Portsmouth, Portsmouth, United Kingdom; National Taiwan Ocean University, TAIWAN

## Abstract

The search for alternative live feed organisms and the progression of Integrative Multi-Trophic Aquaculture (IMTA) are currently being highly prioritised in EU strategies. Caprellids could potentially be an important exploitable resource in aquaculture due to their high levels of beneficial polyunsaturated fatty acids, fast growing nature and widespread distribution. Furthermore, since they are mainly detritivorous, they could be excellent candidates for integration into IMTA systems, potentially benefitting from uneaten feed pellets and faeces released by cultured fish in fish farms and sea-cage structures. Despite this, there is a lack of experimental studies to: (i) test inexpensive diets for caprellids, such as detritus, (ii) develop sustainable caprellid culture techniques and (iii) include caprellids in IMTA systems. The main aim of this study was to determine whether detritus (D) in the form of fish faeces provided an adequate diet for caprellids in comparison to other traditional diets, such as *Artemia* nauplii (A) or phytoplankton (P). Adult survival rate was shown to be significantly higher for caprellids fed with D. Conversely, hatchlings had the highest survival rate with A, although the juvenile growth rate and number of moults was similar in the three diets. With regard to lipid composition, caprellids fed with A had higher concentrations of Triacylglycerols (TAG) and Phosphatidylcholine (PC) while those fed with P or D were richer in polyunsaturated fatty acids, especially 22:6(n-3) (DHA). Interestingly, caprellids fed with D were also a rich source of 18:2(n-6) (LA), considered to be an essential fatty acid in vertebrates. It was found that detritus based mainly on fish faeces and uneaten feed pellets can be considered an adequate feed for adult caprellids, providing a source of both omega-3 (DHA) and omega-6 (LA) fatty acids. Hatchlings however seem to require an additional input of TAG and PC during juvenile stages to properly grow.

## Introduction

Aquaculture accounts for nearly 40% of fin- and shellfish consumed worldwide, reaching 62.7 million tonnes in 2011 [1]. To meet future demands, aquaculture production will need to more than double to 140 million tonnes by the year 2050 [2]. The challenges facing aquaculture are increasingly recognised by the European Commission and are addressed through the EU Blue Growth Strategy and the reformed Common Fisheries Policy [1]. Additionally, aquaculture is given full consideration in all European strategies for Marine Biotechnology [3].

In the framework of the innovative research programmes in aquaculture there are currently two areas of increasing interest: (i) the search for alternative live feed organisms, and (ii) the progress in ‘Integrated Multi-Trophic Aquaculture’ (IMTA). (i) Many marine finfish aquaculture efforts, particularly for larval or juvenile finfish stages, utilise a limited range of live feed organisms such as: *Artemia*, rotifers, copepods and mysid shrimp ([4] and references therein). Although formulated diets are being developed to replace these organisms (and thus reduce their production cost and support), live feed organisms remain vital in aquaculture as better results are obtained from their use [5, 6, 7]. Consequently, there is an urgent need to explore and investigate the potential of novel aquatic organisms as live feed in aquaculture. (ii) A number of methods to increase production levels in aquaculture have attracted attention, such as offshore aquaculture installations [8], recirculating aquaculture systems [9], and especially the innovative technology IMTA [10]. IMTA involves the integrated cultivation of fed species (e.g. finfish) together with extractive species (marine invertebrates and/or algae) which feed on detritus from the fed species [1]. IMTA allows species from two or more trophic levels to grow simultaneously in the same farm, with the waste of one feeding the other [11]. It has been demonstrated that macroinvertebrate fauna present in fouling communities can take up sinking organic matter from fish farms and sea-cage structures, benefitting from uneaten feed pellets and faeces from the cultured fish [12,13]. Along with improving effluent management, IMTA approaches reduce waste, diversify products, improve the economics, expand the range of suitable development sites and increase the biosecurity of farms ([14] and references therein). The use of IMTA in combination with biofloc technology has also been suggested to improve culture efficiency and water quality [15]. If proven to be less harmful to the environment, finfish produced within these systems could potentially be marketed as ‘environmentally friendly’ products [1]. In the future, IMTA could become an integral part of coastal regulatory and management frameworks, with the challenge being the establishment of safe and stable systems with an economically feasible output [3, 16].

Amphipod crustaceans are among the most diverse group of crustaceans with respect to life styles, trophic types, habitats and sizes [17]. Amphipods inhabit a variety of marine environments and consequently show a high diversity of feeding habits [18]. Due to their nutritional characteristics, amphipods could serve as an adequate alternative live or dead feed resource for aquaculture [7,19,20]. During recent years, several promising results have been obtained by using marine amphipods as alternative prey for fishes [21, 22] and cephalopods [23, 24] and several amphipod species have recently been cultured under controlled conditions for potential use in aquaculture (e.g. [25]). [4] examined aspects of known biology and ecology of caprellid amphipods and their potential suitability as a novel marine finfish feed, concluding that caprellids were worthy of consideration for application in marine finfish aquaculture. The suggestion was based on the following characteristics: (i) they have a widespread global distribution, (ii) they form an important natural dietary component in a variety of coastal marine finfish, (iii) they contain high levels of beneficial polyunsaturated fatty acids, (iv) caprellids are relatively sedentary, readily colonise artificial structures (fouling communities) and under appropriate conditions can reach high biomass, especially around fish farms, (v) they exhibit fast growth with several generations per year, (vi) they are opportunistic feeders, (vii) some species show wide environmental tolerances, and (viii) recent studies have indicated the potential suitability of caprellids to larger scale culture (i.e. [25]).

**Fig 1:**
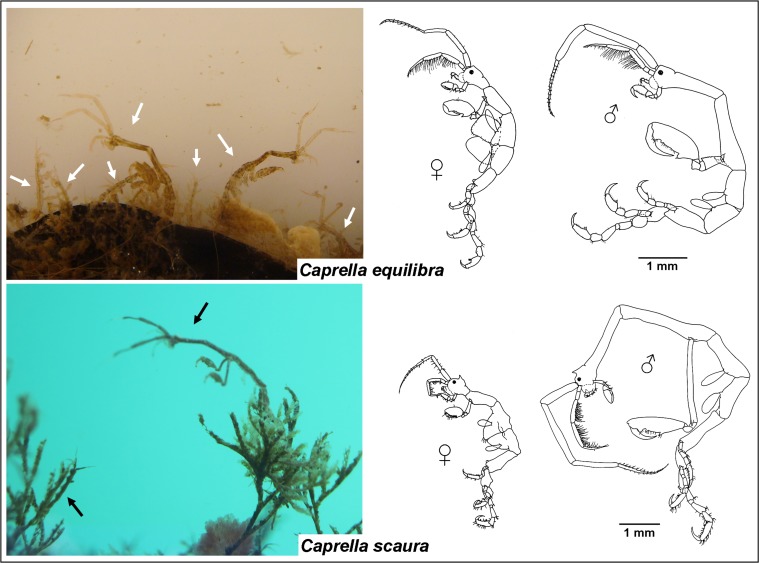
Caprellid species used in the present study. Left: field populations inhabiting marinas of Southern Spain. Right: Lateral view figures of male and female specimens of each species.

Despite the consensus in considering caprellids as a potential resource in aquaculture, field work and experimental approaches are very scarce in this regard. There is still a lack of studies (e.g. cuttlefish: [26]) in connection with the use of caprellids as alternative feed (live or dehydrated) for fishes, mollusks or crustaceans. Provided that caprellids feed mainly on detritus [27], they could be excellent candidates for integration into IMTA systems. Caprellids could have a beneficial role to play in IMTA as a combined bioremediator, feed resource and macroalgal enhancer [4]. Caged mariculture typically releases particulate material (detritus mainly composed of faeces and uneaten fishfeed pellets) which can support caprellid populations [13,28]. Marine fish farms provide substratum and nutrients for caprellids, and caprellids in turn may provide a bioremediation mechanism for fish farms, as well as an exploitable feed resource [4]. There are no experimental studies exploring this potential however, and urgent outstanding issues are to test different diets for rearing caprellids (especially inexpensive ones such as detritus) and to develop and integrate economically sustainable caprellid culture techniques into IMTA systems. As a first step in understanding the usefulness of caprellids in IMTA, it is therefore mandatory to test the suitability of detritus in comparison with other diets (such as *Artemia* nauplii or phytoplankton) as an adequate feed for caprellids in terms of nutritional support and the survival or growth rates achieved. [4] suggested the use of *Caprella equilibra* Say, 1818 as one of the preliminary candidate species to be ideally suited for large-scale intensive land-based culture. This species was selected for the present study, together with *Caprella scaura* Templeton, 1836 (Fig 1). These species are the most common caprellids associated with fouling communities along the Iberian Peninsula (see [29]). In the case of *Caprella scaura*, the large volume of organic detritus in its gut contents suggests that it may play an important role as a vector for carbon transfer from detritus to top predators [30]. *Caprella equilibra* has been established in the Mediterranean and the East Atlantic coast for hundreds of years and can be considered native in this region, although it can also be classified as cryptogenic based on the difficulty of determining its origin [31,32]. In the Iberian Peninsula, *Caprella scaura* is a non-native species. It was recorded for the first time in 2005 by [33] in Girona. Currently, this species is spreading quickly along marinas of the Mediterranean and East Atlantic, reaching high densities and competitively displacing *C*. *equilibra* [29]. [34] and [35] found strong morphological and molecular evidence that *C*. *scaura typica* and *C*. *scaura scaura* correspond to the same “variety” and that this “variety” is the only one expanding its distribution range with a strong invasive capacity. This is the “variety” widely distributed in the Iberian Peninsula, which has been used as a target species in this study. Both species are easy to collect and can be found in high densities on artificial structures (floating pontoons, buoys, ropes, etc.). Recent studies have revealed that *C*. *equilibra* has a high nutritional value potentially significant for aquaculture purposes, with high contents of polyunsaturated fatty acids and polar lipids and proteins [7].

Lipid accumulation is the most widespread long-term energy storage strategy in aquatic crustaceans and their reproductive potential is largely dictated by lipid content [36]. Of the Lipid Classes (LCs), Triacylglycerol (TAG) is the main storage lipid in benthic amphipods and fatty acids (FAs) are necessary for maintaining cell membrane integrity, lipid transport, pigmentation, and are the building blocks for many hormones (see [4] and references therein). A diet that does not provide adequate amounts of fatty acids can affect the life cycle of crustaceans [37]. For all these reasons, lipid composition (LCs and FAs) was selected as a biomarker of nutritional quality in the present study. As a growing number of studies use FAs as biomarkers at the community level, it has become increasingly important to improve our understanding of the effect of diet on FA profiles of a range of marine invertebrates [38].

The main aim of this study was to determine whether detritus (D) can be considered as an adequate diet for caprellids (*C*. *equilibra* and *C*. *scaura*) in comparison with traditionally used diets, such as *Artemia* nauplii (A) or phytoplankton (P). The general aim was addressed through specific objectives, which were: (i) to explore if the caprellids were able to ingest a variety of diets, including D; (ii) to compare the nutritional values of D with A and P by analysing lipid composition (LCs and FAs), (iii) to determine if the LC and FA composition of caprellids changed in response to different diets (A, P or D), (iv) to examine the influence of diet (A, P or D) on the survival and growth rates of adult and juvenile specimens.

## Material and Methods

### Organisms, study sites and field sampling

Two caprellid amphipods were selected for the present study: *Caprella equilibra* and *Caprella scaura* (see introduction section for details of these species). All the specimens used in the experiment and chemical analysis for this study were collected in June 2015 from two marinas located in Southern Spain. *Caprella equilibra* was sampled from Nuevo Portil Marina, El Rompido, Huelva (37°12’46”N, 7°04’51”W) where it was the dominant amphipod, associated mainly to the bryozoans *Bugula neritina* and *Zoobotryon verticillatum*, and the hydroid *Ectopleura crocea*. *Caprella scaura*, absent in Nuevo Portil Marina, was collected from colonies of *B*. *neritina* from La Línea Marina -Club Marítimo Linense, Cádiz (36°09’36”N, 5°21’34”W). No specific permissions were required for collections in these marinas, and the field studies did not involve endangered or protected species. In previous samplings conducted in 2011, La Línea Marina was dominated by *C*. *equilibra* and *C*. *scaura* was absent [32]. In the last 4 years *C*. *scaura* has invaded this marina and reached high densities, displacing *C*. *equilibra*. Colonies of bryozoans and hydroids attached to floating pontoons, buoys and ropes of the marinas were handpicked. Preliminary caprellid sorting was conducted in situ, before the individuals were transported with aeration together with some bryozoan colonies to the laboratory (see [25]).

### Experimental designs in laboratory conditions

#### Experiment 1: Are caprellids able to feed on a variety of diets?

To test the feeding preferences of *C*. *equilibra* and *C*. *scaura* and to verify the ability of caprellids to properly ingest different feed types under laboratory conditions, the following experiment was conducted. 80 adult specimens of each species were isolated in small, 120ml glass containers with a diameter of 6.5cm and a height of 6cm (4 specimens per container). An equal amount of males and females were considered to avoid potential differences among sexes. A 1 mm plastic mesh was used as a substratum for attachment. Specimens were maintained without feed for a duration of 24 hours to empty their guts and the water was then changed to remove faecal pellets. 16 specimens (4 glasses) of each species were then used for each different diet; *Artemia* nauplii (A), phytoplankton (P), detritus (D), the three items together to explore preferences (All) or no additional feed (NF). Trace natural detritus would have entered all containers by attachment to specimens. *Artemia* nauplii were hatched from cysts maintained (1–2 days) in a Brine Shrimp Hatchery Hobby® with seawater at 25°C. The *Artemia* nauplii used for the experiments were not enriched. The phytoplankton used consisted of a mixture of freeze-dried microalgae (Easy Reefs®) containing *Phaeodactylum*, *Tetraselmis* and *Nannochloropsis* (1:1:1, percentage in dry weight). Detritus was obtained by scraping the bottoms of aquaculture tanks used for culture of meagre (*Argyrosomus regius*) at the IFAPA center “El Toruño” experimental aquaculture station (Cádiz, Spain) and consisted primarily of fish faeces and also of uneaten fishfeed pellets used as feed for meagre. Caprellids were fed *ad libitum* and after 24 hours were preserved in 90% ethanol. The caprellids were maintained at 20°C with a photoperiod of 12h light: 12h dark. The seawater used for the culture was treated by filtration (through a 0.45μm Millipore filter, Merk, Spain) and UV irradiation, and had a salinity of 35.5psu. In addition to the specimens used in the 5 treatments of the experimental design, 16 adult specimens of each species were fixed directly after collection from the marinas to analyse the diet in field populations.

For the diet study, individuals were analysed following the methodology of [39] with slight variations, recently proposed to study the diet of amphipods [18, 27]. Specimens of each species were placed in vials with Hertwig’s liquid (consisting on 270g of chloral hydrate, 19ml of chloridric acid 1N, 150ml of distilled water and 60ml of glycerin) and heated in an oven at 65°C for 2 to 10 hours depending on the size of the specimens. After this, they were mounted on slides for study under a microscope. The percentage of the absolute gut content (at 40x or 100x), described as the total area occupied by the content in the whole digestive tract, and the relative gut content (at 100x or 400x), described as the area occupied for each component within the total gut content, were estimated using a microscope equipped with an ocular micrometer.

#### Experiment 2: Does diet influence the lipid composition and survival of adults of *C*. *equilibra* and *C*. *scaura*? Is detritus an adequate feed for them?

Provided that caprellids were able to feed on the three diets considered (see experiment 1), the same diets (A, P or D) were used to test the influence of feed on the lipid composition of the caprellids and therefore their nutritional value for use as a resource for aquaculture. The present experiment was designed to last 12 days, as [38] reported changes in lipid composition of littoral amphipods in relation to diet after 12 days. 18 aquaria (20cm x 13cm x 13cm) containing 3l of seawater (see experiment 1) were used; 3 for each treatment (A vs P vs D) of each species (*C*. *equilibra* vs *C*. *scaura*). In this experiment “All feeds” and “no feed” treatments were not included, mainly for two reasons: (i) in this case, the objective was not to explore feeding selection among items; to clearly evaluate the effect of each diet on the lipid composition it was necessary to use each diet separately. (ii) The use of additional treatments such as a combination of feeds or starvation was prevented due to the high amount of individuals required. The high number of specimens required is a common constraint limiting the number of experimental treatments possible. Forty adult specimens were added to each aquarium equating to a total of 360 specimens of *C*. *equilibra* and 360 specimens of *C*. *scaura*. Folded meshes, as proposed by [25] were used as substratum. Two small stones were used to prevent buoyancy of the meshes, maintaining their position at the bottom of the aquaria. The caprellids were fed *ad libitum* daily and the water of the aquaria was replaced every two days. Any dead specimens, uneaten feed or faecal pellets were removed daily. Aquaria were maintained with a continuous air supply, a water temperature of 20°C, a photoperiod of 12h light: 12h dark, and a seawater salinity of 35.5psu. Twelve days after the introduction of the caprellids in the aquaria, the plastic meshes were removed individually. The number of remaining adult specimens was counted, and the pool of specimens for each aquarium (considered as a replicate) was immediately frozen and stored at -80°C for chemical analysis. Three replicates of the three feed types used for feeding *C*. *equilibra* and *C*. *scaura* during the experiment (A, P and D) were also frozen for chemical analysis to characterise their lipidic nutritional value. In order to compare the treatments under laboratory conditions to the lipid composition of field populations, three replicates (three pools of specimens) for each species (*C*. *equilibra* vs *C*. *scaura*) were frozen immediately after collection from the marinas and stored at -80°C for chemical analysis.

#### Experiment 3: Does diet influence the survival and growth rate of juveniles of *C*. *equilibra* and *C*. *scaura*? Is detritus an adequate feed for them?

Thirty ovigerous females (15 of *C*. *equilibra* and 15 of *C*. *scaura*) taken directly from the marinas were isolated individually in small glass containers of 120ml with a diameter of 6.5cm and a height of 6cm. A 1 mm plastic mesh, replaced every 5 days, was used as a substratum. This experiment was focused on instars I-III of the juvenile stage. As a reference, [40] found the duration of the juvenile period in *C*. *equilibra* after emerging from the brood pouch to be approximately 14–16 days at 20°C, corresponding with instars I-III (from instar IV, sexes can be differentiated). Therefore, all the hatchlings which emerged from each female were monitored during the first 16 days of their life history, corresponding with the juvenile stage. Three treatments were also considered (A vs P vs D) and 5 replicas (consisting of the hatchlings produced by 5 females of each species) were used for each treatment. Juveniles were fed *ad libitum* every day, and water was also replaced daily. All of the juveniles were counted daily under a binocular microscope and observations were made of any signs of moulting or parental care. After 16 days, all of the juveniles were fixed in 90% ethanol. The number of articles of the flagellum of antenna 1, which is indicative of the number of moults (see [26]), were counted in each juvenile. Photographs of each specimen were taken with a Motic K-400L stereomicroscope and measures of the total body length were taken using the software Scion Image Alpha 4.0.3.2 (2000–2001 Scion Corporation).

### Chemical analysis: lipid classes (LCs) and fatty acids (FAs)

Three replicates of the following samples from experiment 2 were used for chemical analysis: (i) the three feed types the caprellids were subjected to (A, P and D), (ii) adult specimens of the two caprellid species collected from marinas and frozen immediately as reference values and (iii) adult specimens of the two caprellid species fed either A, P or D during 12 days. Each replicate consisted of a pool of specimens in order to reach the minimum quantity required for chemical analysis, which was 10mg. Quantities used in other studies usually range from 10-40mg [7,38,41,42] to 0.5–3 g [13]. Unfortunately, the small weight of caprellids prevented the analysis of LCs and FAs in juveniles, since it was not possible to reach the minimum required weight for the chemical procedure, even when all the individuals of all the replicates of each treatment were pooled. LCs and FAs were therefore analysed only for adult specimens. Taking into account that [36] found differences among sexes in some lipid classes, and that [41] showed sex differentiation using fatty acid signatures, the same weight of male and female specimens were considered for each replicate to avoid the influence of sex in the results.

Samples were freeze-dried for 24h at -50°C. The lipid fraction was extracted according to the Folch-Lee method [43]. Total lipid was extracted with chloroform:methanol (2:1v/v) containing 0.01% of butylated hydroxytoluene (BHT) as an antioxidant [44]. The organic solvent was evaporated under a stream of nitrogen and the lipid content was determined gravimetrically. Lipid classes were separated by one-dimensional double development high-performance thin-layer chromatography (HPTLC) using methyl acetate/isopropanol/chloroform/methanol/0.25% (w/v) KCl (25:25:25:10:9 by volume) as the polar solvent system and hexane/diethyl ether/glacial acetic acid (80:20:2 by volume) as the neutral solvent system. Final quantification of lipid classes was made by densitometry in a CAMAG scanner at a wavelength of 325nm, and by comparison with external standard (Sigma-Aldrich) (see [45]). For fatty acid analysis, total lipid extracts were subjected to acid catalysed transmethylation for 16 hours at 50°C, using 1mL of toluene and 2mL of 1% sulphuric acid (v/v) in methanol. The resulting fatty acid methyl esters (FAME) were separated and quantified using a Shimadzu GC 2010-Plus (Shimadzu) gas chromatograph equipped with a fame-ionisation detector (280°C) and a fused Tecnokroma–Suprawax-280^TM^ (15 m x 0.1 mm I.D.). Hydrogen was used as carrier gas and the oven initial temperature was 100°C, followed by an increase at a rate of 20°C min^-1^ to a final temperature of 250°C for 8 min. Individual FAME were identified by reference to authentic standards and to a well-characterised fish oil.

### Statistical analysis

To determine whether the amount of ingested feed varied among species and treatments (experiment 1), a two-way ANOVA was conducted with the following factors: ‘Species’ which was a fixed factor with two levels: *C*. *equilibra* (Eq) and *C*. *scaura* (Sc), and ‘Treatment’ which was a fixed factor orthogonal with ‘Species’ consisting of six levels: Marinas (caprellids taken directly from the marinas), A (caprellids fed with *Artemia*), P (caprellids fed with phytoplankton), D (caprellids fed with detritus), all feeds (caprellids fed with the three diets simultaneously) and no feed (caprellids given no feed).

The differences in survival rates of adult males (experiment 2) and juveniles (experiment 3) among species and treatments were also tested using two-way ANOVA, with factor ‘Species’ (Eq vs Sc) and factor ‘Treatment’ (A vs P vs D).

For testing differences in the length of juveniles and number of flagellar articles in antenna 1 (experiment 3) among treatments, one-way ANOVAs were used for each species separately, with the single factor treatment (A vs P vs D) since the total mortality of juveniles of *C*. *equilibra* fed with D prevented the use of two-way ANOVAs.

Two-way ANOVAs were also used to compare the main lipid classes and fatty acids (experiment 3) among species (Eq vs Sc) and treatments (A vs P vs D). Previously one-way ANOVA was also conducted to explore differences in lipid composition among the three types of feed used in the experiment for feeding caprellids.

Prior to ANOVAs, the homogeneity of variances was tested with Cochran’s C-test. Where variances remained heterogeneous even after data transformation, untransformed data were still analysed, as ANOVA is a robust statistical test and is relatively unaffected by the heterogeneity of variances, particularly in balanced experiments [46]. In such cases, to reduce type I error, the level of significance was reduced to <0.01. Where ANOVA indicated a significant difference for a given factor, the source of difference was identified using Student-Newman-Keul (SNK) tests [46].

Principal Component Analyses (PCAs) were carried out to show the relationship among treatments, feeds (diets) and samples from marinas (reference values) according to the lipid classes and fatty acid matrices. Differences in fatty acid and lipid classes composition among species and diets were tested by the use of a permutational multivariate analysis of variance (PERMANOVA) with two factors: ‘species’, a fixed factor with two levels (Eq vs Sc) and ‘treatment’, a fixed factor orthogonal with ‘species’ consisting of three levels (A vs P vs D). Analysis was based on Euclidean distance measures and Monte Carlo tests were included. Significant P-values were obtained by computing 9999 permutations of residuals under a reduced model as this method gives the most accurate Type I error for complex designs [47]. Pairwise comparisons were then used. Additionally, to test the dispersion among samples for the factors ‘species’ and ‘treatments’, a permutational analysis of multivariate dispersions (PERMDISP) was used.

Univariate analyses were conducted with GMAV5 [48] and multivariate analyses were carried out using the PRIMER v.6 plus PERMANOVA package [49].

## Results

### *Caprella equilibra* and *C*. *scaura* as opportunistic feeders (Experiment 1)

**Fig 2:**
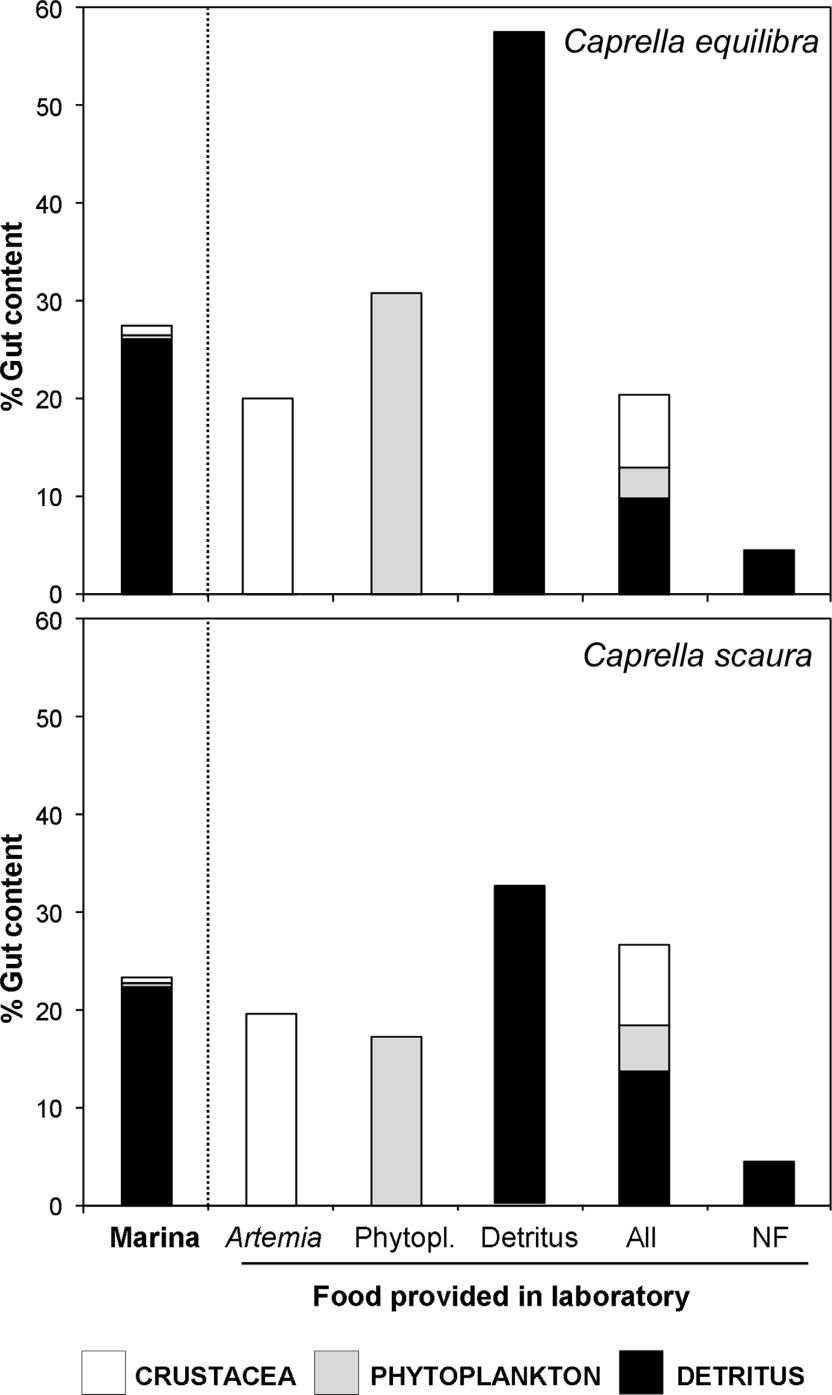
Total area occupied by the gut content in the whole digestive tract of *Caprella equilibra* and *C*. *scaura*. ‘Marina’ includes data of the field populations directly fixed after collection. Treatments represent the different feed provided in laboratory conditions: *Artemia* nauplii, Detritus, Phytoplankton, All feed provided together (All), and no feed provided (NF). The bars indicate the percentage of each item observed in the gut under the microscope (Crustacea, Phytoplankton and Detritus). The Crustacea are represented by copepods in specimens from the field populations of the marinas and *Artemia* in those maintained in laboratory conditions fed with *Artemia*.

Gut content of the specimens of *C*. *equilibra* and *C*. *scaura* which were directly fixed following collection in the marinas consisted mainly of detritus (more than 95% of the total), and included crustaceans (harpacticoid copepods) and phytoplankton (microalgae) (Fig 2). In laboratory conditions, when caprellids were offered a variety of diets, all items were observed in the gut, showing the ability of caprellids to ingest all three types of feed (A, P and D). When the three feed items were provided simultaneously, both caprellid species showed all three of them in their guts (Fig 2). Caprellids were able to actively prey on the live *Artemia nauplii*. In the control treatment ‘No feed’, a small percentage of detritus (less than 5%) was registered in the gut, indicating that specimens isolated in the glass containers ingested the detritus attached to their body surface. When detritus was the single item provided, the amount of feed ingested (measured as the total area occupied by the gut content in the whole digestive tract) was significantly higher than in other treatments (Fig 2, Table 1). There were no significant differences in the quantity ingested among specimens fed with P, A or a combined diet of A+P+D (Table 1).

**Table 1:** Results of the two-way ANOVA for the percentage of gut contents during the feeding experiment.

Source of variation	df	MS	F	*P*	F versus
Species (Sp)	1	2352.00	3.28	0.0720	Res
Treatment (Tr)	5	5802.99	8.08	0.0000***	Res
Sp x Tr	5	998.02	1.39	0.2300	Res
Residual (Res)	180	717.98			
Cochran's test		C = 0.16 n.s.			
Transformation		None			
SNK		D>(Ma = A = P = D = All)>NF			

***P<0.001. Ma = Marinas, A = *Artemia*; P = phytoplankton; D = detritus; All = All feed; NF = No feed.

### Lipid composition of the diets used in the experiments

The three diets each had a distinct LC composition (Table 2) and FA profiles (Table 3). In connection with LCs, *Artemia* nauplii were characterised by a higher content in PC (one-way ANOVA, F = 322.1, p<0.001) and TAG (one-way ANOVA, F = 1125.5, p<0.001), while detritus was richer in ST (one-way ANOVA, F = 6.6, p<0.05) and phytoplankton had a low content of most of the lipid classes analysed. In regard to FA, the saturated 16:0, the monounsaturated 16:1, OA, 18:1(n-7) and the polyunsaturated EPA were the dominant components in the three diets. However, the percentage of FA significantly differed among diets. Detritus had a higher content of 16:0 (one-way ANOVA, F = 307.1, p<0.001), 18:0 (one-way ANOVA, F = 1454.4 p<0.001), LA (one-way ANOVA, F = 1157.1, p<0.001) and DHA (one-way ANOVA, F = 620.1, p<0.001), but a lower content of 16:1 (one-way ANOVA, F = 3562.9, p<0.001). *Artemia* nauplii were significantly richer in 18:1(n-7) (one-way ANOVA, F = 1125.5, p<0.001) and EPA (one-way ANOVA, F = 4563.4 p<0.001) while the phytoplankton had a significantly higher content of LNA than *Artemia* or detritus (one-way ANOVA, F = 3237.4, p<0.001).

**Table 2:** Main lipid classes (μg/100μg DW) of the three types of feed used in the experiment as diets (A: *Artemia*, P: Phytoplankton, D: Detritus), the caprellids (Eq: *C*. *equilibra*, Sc: *C*. *scaura*) collected from marinas (used as reference values) and the caprellids fed either *Artemia*, phytoplankton or detritus during 12 days in laboratory conditions (treatments).

	FEED			CAPRELLIDS							
	Diets			Marinas		Treatments					
	A	P	D	Eq	Sc	EqA	EqP	EqD	ScA	ScP	ScD
Phosphatidylcholine (PC)	1.00	0.53	0.07	3.93	2.92	2.35	1.76	1.87	2.54	2.26	1.25
Phosphatidylethanolamine (PE)	0.40	-	0.15	1.45	1.13	0.95	0.73	0.85	0.94	0.98	0.58
Phosphatidylinositol (PI)	0.15	0.11	0.28	0.18	0.21	0.19	0.15	0.15	0.17	0.20	0.11
Phosphatidylserine (PS)	-	-	0.43	0.21	0.18	0.18	0.20	0.25	0.16	0.26	0.15
Triacylglycerols (TAG)	4.12	-	1.60	3.18	2.40	3.01	0.44	0.41	5.43	1.07	0.57
Sterols (ST)	0.04	0.01	1.68	0.27	0.28	0.15	0.29	0.19	0.22	0.21	0.12

**Fig 3:**
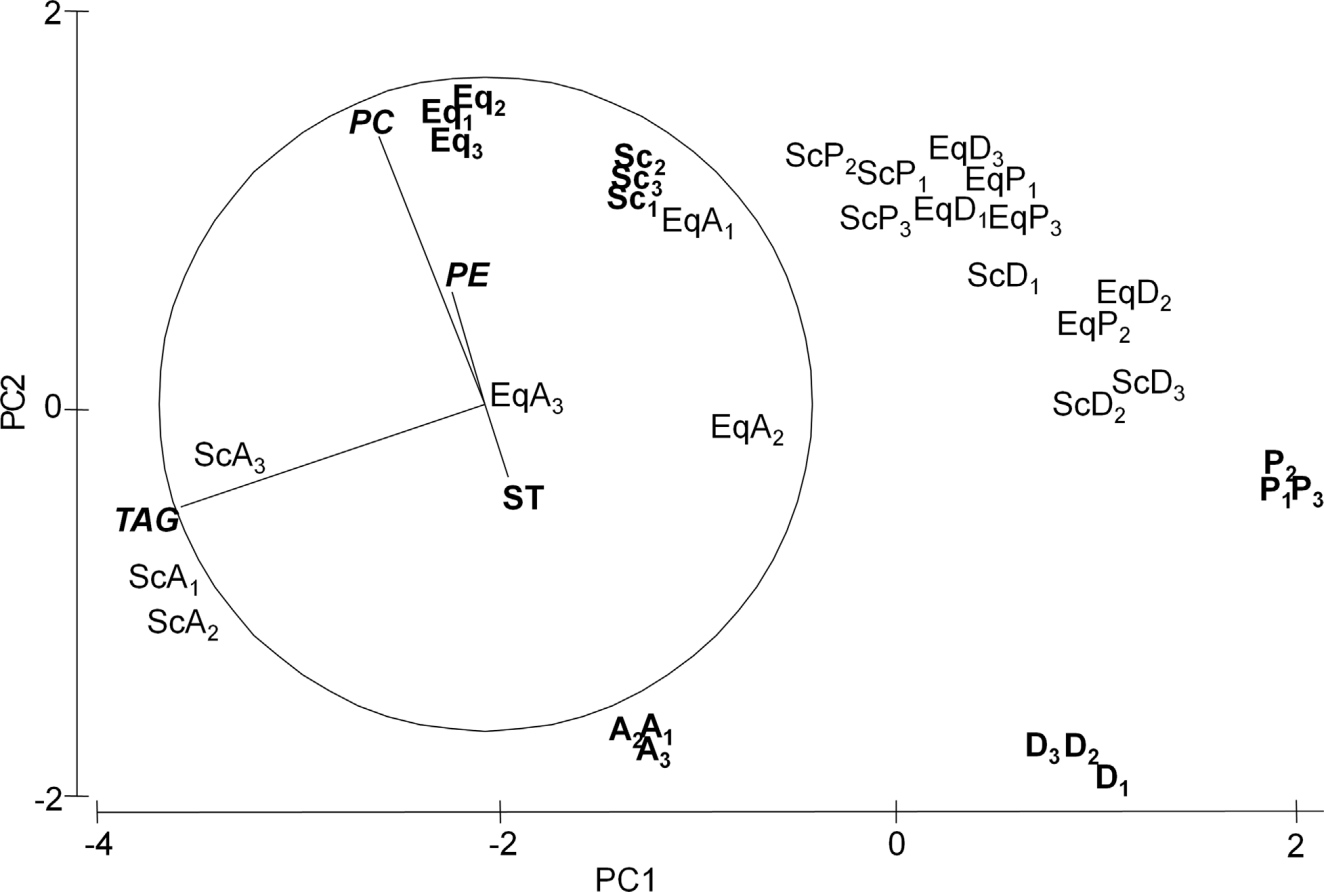
Principal Component Analysis (PCA) plot based on the Lipid Classes (LCs) composition. LCs were analysed for the three types of feed used in the experiment as diets (A: *Artemia*, P: Phytoplankton, D: Detritus), the caprellids (Eq: *C*. *equilibra*, Sc: *C*. *scaura*) collected from marinas (used as reference values) and the caprellids fed either *Artemia*, phytoplankton or detritus for 12 days under laboratory conditions (EqA, EqP, EqD, ScA, ScP, ScD). Subscript numbers (1,2,3) correspond to each replicate value.

Values are mean of 3 replicates. For caprellids, each replicate consisted of a pool of adult specimens. Dispersion among replicates can be observed in PCA (Fig 3).

**Table 3:** Fatty acid composition (expressed as % of the total identified fatty acids) of the three types of feed used in the experiment as diets (A: *Artemia*, P: Phytoplankton, D: Detritus), the caprellids (Eq: *C*. *equilibra*, Sc: *C*. *scaura*) collected from marinas (used as reference values) and the caprellids fed either *Artemia*, phytoplankton or detritus during 12 days in laboratory conditions (treatments).

	FEED			CAPRELLIDS							
	Diets			Marinas		Treatments					
	A	P	D	Eq	Sc	EqA	EqP	EqD	ScA	ScP	ScD
*Saturated (SFA)*											
14:0	1.84	4.06	2.42	1.82	3.17	0.93	0.70	0.53	0.81	0.63	0.62
15:0	0.43	1.20	0.68	0.53	0.65	0.28	0.85	0.46	0.39	0.75	0.60
16:0	12.33	19.44	27.58	22.06	19.58	13.80	20.06	18.96	12.60	17.71	17.85
17:0	0.92	-	0.93	1.37	1.55	0.99	1.73	0.98	1.05	2.14	1.27
18:0	4.91	0.64	9.44	6.10	5.31	4.52	5.56	4.94	4.01	5.29	4.91
20:0	-	-	1.10	0.12	0.53	-	-	0.09	0.15	0.19	0.17
22:0	0.34	-	0.98	0.05	0.05	0.19	-	0.09	0.18	-	-
23:0	-	-	-	-	-	0.19	-	-	0.43	0.94	0.89
24:0	-	0.77	0.97	0.09	-	-	0.21	-	0.02	-	-
*Total SFA*	20.77	26.11	44.09	32.15	30.84	20.90	29.10	26.05	19.65	27.63	26.31
*Monounsaturated (MUFA)*											
16:1	19.15	17.75	3.04	2.79	4.87	10.32	1.75	1.03	10.71	2.73	1.87
17:1	1.56	4.70	-	-	-	0.52	-	-	1.17	-	0.22
18:1(n-9) (OA)	17.58	3.45	20.49	9.41	10.08	21.76	14.06	18.17	20.52	11.68	18.34
18:1(n-7)	12.91	2.05	3.78	1.78	2.50	10.89	4.46	2.89	11.59	4.46	4.20
20:1(n-11)	-	-	0.11	0.08	-	-	-	-	-	-	-
20:1(n-9)	0.49	0.40	1.38	1.35	1.47	1.65	1.78	2.11	1.58	1.69	2.27
22:1(n-11)	-	-	0.82	-	-	-	-	-	-	-	-
22:1(n-9)	-	-	-	0.15	0.06	0.24	-	0.06	0.18	0.15	0.18
24:1	-	-	1.04	-	-	0.29	-	-	0.50	-	-
*Total MUFA*	51.69	28.37	30.66	15.56	18.97	45.68	22.05	24.26	46.25	20.70	27.08
*Polyunsaturated (PUFA)*											
16:2(n-4)	1.11	2.70	-	0.15	0.31	0.42	-	-	0.45	-	-0
16:3(n-4)	0.38	2.09	-	0.11	0.09	-	-	-	-	-	-
16:4(n-1)	0.06	0.79	-	-	0.11	-	-	-	-	-	-
18:2(n-6) (LA)	2.92	4.41	16.46	1.79	2.06	3.38	2.09	7.66	2.99	2.60	9.69
18:2(n-4)	0.45	-	-	-	0.03	0.14	-	-	0.22	-	-
18:3(n-6)	0.59	0.23	-	0.12	0.39	0.40	-	-	0.37	0.07	0.06
18:3(n-4)	0.52	-	-	-	-	0.31	-	-	0.43	-	-
18:3(n-3) (LNA)	4.27	20.79	1.95	1.41	1.09	3.87	1.32	0.41	3.07	2.41	0.76
18:4(n-3)	1.41	1.37	-	1.92	2.05	0.78	-	-	0.75	0.46	0.26
20:2(n-6)	-	-	-	1.06	0.77	0.42	0.64	1.50	0.33	0.97	1.78
20:3(n-6)	-	-	-	0.07	0.06	-	-	-	0.07	-	-
20:4(n-6) (ARA)	1.15	1.32	0.28	3.24	4.05	2.02	4.37	4.35	2.39	5.77	4.22
20:3(n-3)	-	0.54	0.15	0.69	0.87	0.66	1.38	1.02	0.73	1.91	0.74
20 4(n-3)	0.43	-	-	0.80	1.07	0.34	-	-	0.27	0.07	0.08
20:5(n-3) (EPA)	14.23	11.28	1.62	19.64	21.11	16.60	20.84	16.28	18.37	21.34	14.39
21:5(n-3)	-	-	-	0.39	0.38	-	0.26	-	0.03	0.18	-
22:4(n-6)	-	-	-	0.09	0.17	-	-	-	-	0.07	-
22:5(n-6)	-	-	-	0.55	0.51	-	0.32	0.64	0.04	1.08	0.89
22:5(n-3) (DPA)	-	-	0.26	0.78	0.82	0.12	0.74	0.91	0.16	0.78	0.59
22:6(n-3) (DHA)	-	-	4.52	19.50	14.25	3.97	16.89	16.91	3.42	13.94	13.13
*Total (n-3)*	20.34	33.98	8.50	45.13	41.64	26.35	41.43	35.52	26.82	41.09	29.96
*Total (n-6)*	4.67	5.96	16.75	6.90	8.01	6.21	7.42	14.16	6.19	10.58	16.65
*Total PUFA*	27.54	45.52	25.25	52.29	50.19	33.43	48.84	49.68	34.10	51.66	46.61

**Fig 4:**
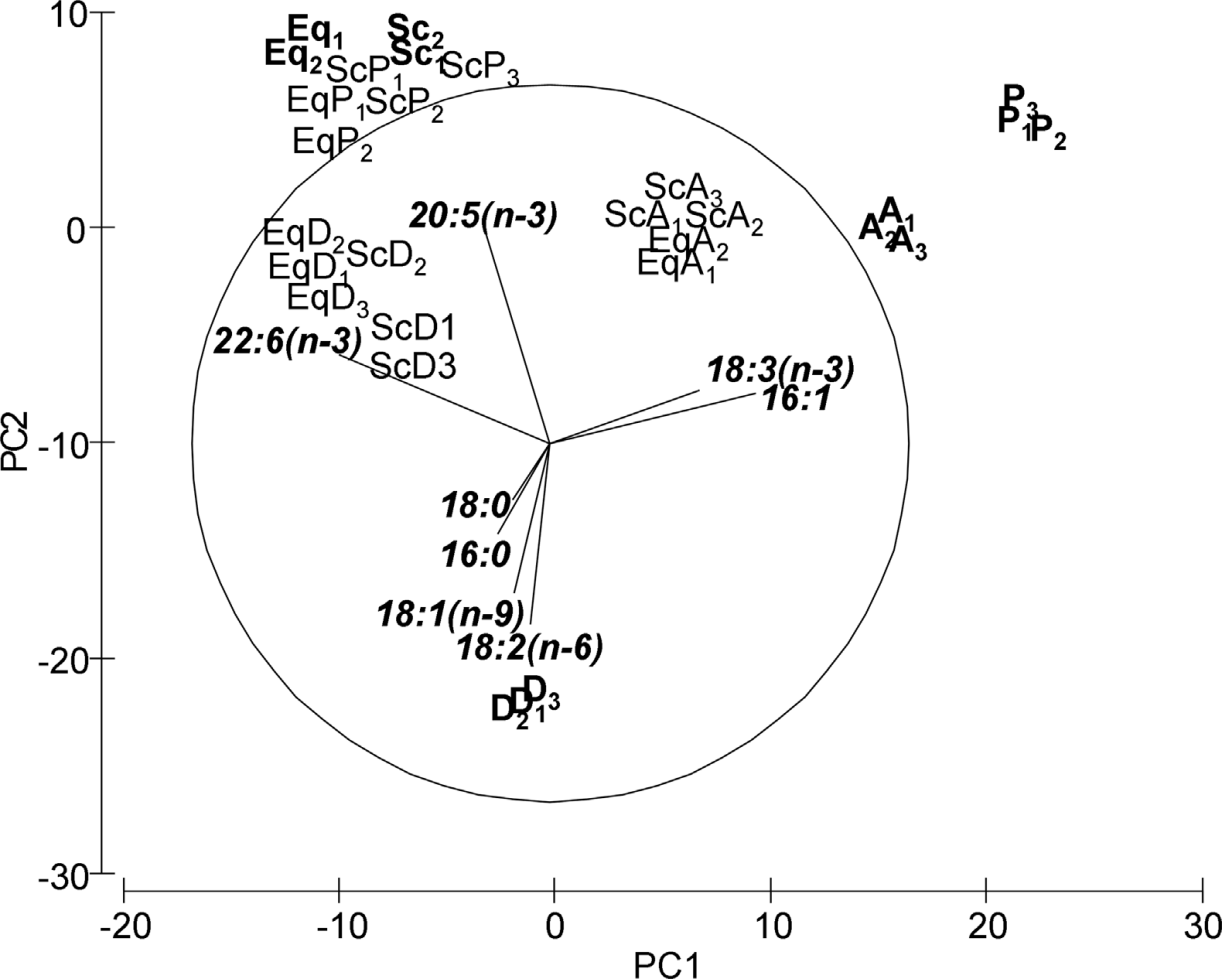
Principal Component Analysis (PCA) plot based on the Fatty Acid (FAs) composition. FAs were analysed for the three types of feed used in the experiment as diets (A: *Artemia*, P: Phytoplankton, D: Detritus), the caprellids (Eq: *C*. *equilibra*, Sc: *C*. *scaura*) collected from marinas (used as reference values) and the caprellids fed either *Artemia*, phytoplankton or detritus for 12 days under laboratory conditions (EqA, EqP, EqD, ScA, ScP, ScD). Subscript numbers (1,2,3) correspond to each replicate value. Replicates Eq3, EqA3, EqP3 and Sc3 are not included since they were damaged during the chemical procedure and lost. For PERMANOVA analyses, the values of these replicates were filled with the mean values of the other two remaining replicates.

Values are mean of 3 replicates. For caprellids, each replicate consisted of a pool of adult specimens. Dispersion among replicates can be observed in PCA (Fig 4). OA: Oleic acid; LA: Linoleic acid; LNA: Alpha linoleic acid; ARA: Arachidonic acid; EPA: Eicosapentanoic acid; DPA: Docosapentanoic acid; DHA: Docosaheanoic acid.

### Influence of the diet on lipid composition and survival rate of adult specimens (Experiment 2)

Both caprellid species showed similar results in the experiment. LC and FA composition of the three treatments considered (A, F and D) are included in Tables 2 and 3, together with the reference values obtained from specimens of field populations (marinas).

PCA results for LCs (Fig 3) showed differences among treatments, and these differences were also reflected by the PERMANOVA (Table 4). Although an interaction between the factors ‘Species’ and ‘Treatment’ was detected, the pair-wise tests of the interaction reflected an identical pattern for both species: the caprellids fed with *Artemia* differed from those fed with phytoplankton or detritus, as shown by the PCA analysis. Axis 1 (Fig 3) explained 72% of the total variance and correlated with TAG. It clearly separated the samples of caprellids fed with *Artemia* (higher content in TAG) from those fed with phytoplankton and detritus (lower content in TAG). Axis 2 explained 22% of the total variance and correlated primarily with PC and secondarily with PE and ST. Interestingly, in only 12 days (duration of experiment), adult specimens of both species fed with the three diets (EqA, EqP, EqD, ScA, ScP, ScD) changed considerably in their LC composition (Eq, Sc). As expected, each treatment produced results close to the corresponding diet (A, P or D) at the end of the experiment, as can be observed in the PCA output (Fig 3). The two-way ANOVA analyses confirmed significant differences in the concentration of TAG and PC among treatments (Table 5). Both variables showed the highest correlation with PCA axis 1 (r = -0.93, p<0.001) and axis 2 (r = 0.86, p<0.001) respectively.

**Table 4:** Summary of the two-way PERMANOVA results examining the lipid classes and fatty acids of caprellids fed either *Artemia* (A), phytoplankton (P) or detritus (D) during 12 days in laboratory conditions.

Source of variation	df	MS	Pseudo-F	*P* (MC)	MS	Pseudo-F	*P* (MC)
		Lipid classes			Fatty acids		
Species (Sp)	1	5.17	12.28	0.0035**	28.92	7.03	0.0078**
Treatment (Tr)	2	27.29	64.81	0.0001***	636.94	154.85	0.0001***
Sp x Tr	2	2.74	6.52	0.0076**	14.25	3.46	0.0324**
Residual (Res)	12	0.42			4.11		
PERMDISP	(Sp)	F = 7.52, *P* = 0.0925 n.s			F = 0.11, *P* = 0.7829 n.s		
	(Tr)	F = 10.10, *P* = 0.0053**			F = 8.67, *P* = 0.0060**		
Pair-wise tests	Tr (Sp)				Tr (Sp)		
Sp x Tr:	*C*. *equilibra*: A≠P; A≠D; P = D				*C*. *equilibra*: A≠P; A≠D; P≠D		
	*C*. *scaura*: A≠P; A≠D; P = D				*C*. *scaura*: A≠P; A≠D; P≠D		

**P<0.01

***P<0.001.

PERMDISP results for the factors “Species” and “Treatment” are also included. MS = Mean Square; MC = Montecarlo.

**Table 5:** Summary of the two-way ANOVA results examining the selected lipid classes and fatty acids (those which correlated with axis 1 and 2 of the PCA analysis). Caprellids were fed either *Artemia* (A), phytoplankton (P) or detritus (D) during 12 days in laboratory conditions.

Source of variation	df	MS	F	*P*	MS	F	*P*	MS	F	*P*
		**Phosphatidylcoline (PC)**			**Phosphatidylethanolamine (PE)**			**Triacylglycerols (TAG)**		
Species (Sp)	1	0.01	0.01	0.9061	0.00	0.01	0.9275	5.16	28.45	0.0002***
Treatment (Tr)	2	1.18	7.09	0.0093**	0.08	1.40	0.2838	25.98	143.20	0.0000***
Sp x Tr	2	0.49	2.98	0.0888	0.09	1.66	0.2313	2.14	11.84	0.0014**
Residual (Res)	12	0.16			0.06			0.18		
Cochran's test		C = 0.26 n.s.			C = 0.53 n.s.			C = 0.57 n.s.		
Transformation		None			None			None		
SNK		A = P; A≠D; P = D			A = P = D			(Sp x Tr)	Eq:A≠P; A≠D; P = D	
									Sc:A≠P; A≠D; P = D	
		**Sterols (ST)**			**16:0**			**18:0**		
Species (Sp)	1	0.00	0.59	0.4558	3.04	11.96	0.0047**	0.05	0.33	0.5740
Treatment (Tr)	2	0.00	0.17	0.8480	27.01	106.22	0.0000***	0.53	3.32	0.0711
Sp x Tr	2	0.02	4.34	0.0382	0.48	1.90	0.1919	0.21	1.33	0.3010
Residual (Res)	12	0.01			0.25			0.15		
Cochran's test		C = 0.63 (p<0.01)			C = 0.54 n.s.			C = 0.78(p<0.01)		
Transformation		None			None			None		
SNK		A = P = D			A≠P; A≠D; P = D			A = P = D		
		**16:1**			**18:1(n-9)**			**18:2(n-6)**		
Species (Sp)	1	1.72	7.96	0.0154*	1.78	1.92	0.1911	2.61	3.13	0.1020
Treatment (Tr)	2	125.57	579.88	0.0000***	110.71	118.98	0.0000***	52.49	62.94	0.0000***
Sp x Tr	2	0.26	1.20	0.3342	2.69	2.90	0.0940	2.46	2.95	0.0980
Residual (Res)	12	0.21			0.93			0.83		
Cochran's test		C = 0.37 n.s.			C = 0.81 (p<0.01)			C = 0.92 (p<0.01)		
Transformation		None			None			None		
SNK		A≠P; A≠D; P≠D			A≠P; A≠D; P≠D			A = P; A≠D; P≠D		
		**18:3(n-3)**			**20:5(n-3)**			**22:6(n-3)**		
Species (Sp)	1	0.14	3.05	0.1062	1.51	2.48	0.1411	11.58	24.42	0.0003***
Treatment (Tr)	2	10.45	221.51	0.0000***	26.89	44.11	0.0000***	175.06	369.00	0.0000***
Sp x Tr	2	1.21	25.83	0.0000***	2.99	4.91	0.0277*	1.61	3.40	0.0677
Residual (Res)	12	0.04			0.60			0.47		
Cochran's test		C = 0.60 n.s.			C = 0.57 n.s.			C = 0.40 n.s.		
Transformation		None			None			None		
SNK		(Sp x Tr)	Eq:A≠P; A≠D; P≠D		(Sp x Tr)	Eq:A≠P; A≠D; P≠D		A≠P; A≠D; P = D		
			Sc:A≠P; A≠D; P≠D			Sc:A≠P; A≠D; P≠D				

*P<0.05

**P<0.01

***P<0.001.

Regarding FA profiles, both PCA and PERMANOVA showed differences between treatments (Fig 4, Table 4). In this case, although treatments also changed the initial FA composition, the phytoplankton treatment (EqP and ScP) showed higher similarities with the field populations inhabiting marinas (Eq and Sc). Unlike for LCs, in which each treatment was closer to its corresponding diet, for FAs the three diets (A, P and D) appeared clearly separated from the treatments. Although an interaction between the factors ‘Species’ and ‘Treatment’ was also detected in the PERMANOVA, the pair-wise tests of the interaction reflected an identical pattern for both species: significant differences between the three treatments. PCA axis 1 (Fig 4) explained 47% of the total variance and correlated with 16:1, 18:3(n-3) (LNA) and 22:6(n-3) (DHA). Axis 2 explained 27% of the total variance and correlated with 16:0, 18:0, 18:1(n.9) (OA), 18:2(n-6) (LA) and 20:5(n-3) (EPA). The two-way ANOVA analyses confirmed significant differences of the selected fatty acids (those which correlated with axis 1 and 2 of the PCA) among treatments, except for 18:0 (Table 5).

**Fig 5:**
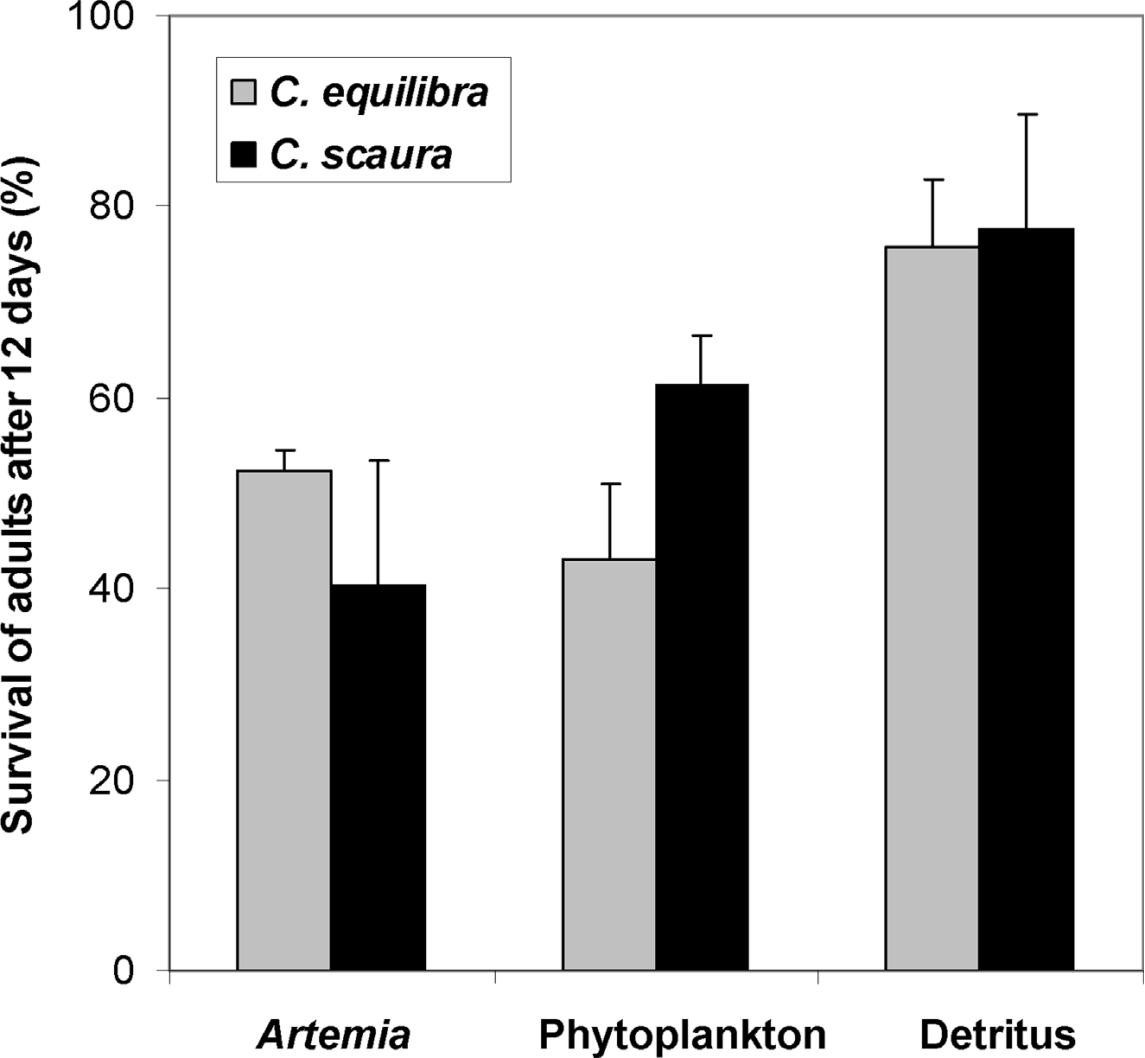
Survival rate (%) of adult specimens of *Caprella equilibra* and *C*. *scaura* after 12 days. Specimens were fed with Artemia, phytoplankton or detritus.

The survival rates of adults after 12 days significantly differed among treatments. For both species, the highest values for survival were obtained from caprellids fed with detritus (Fig 5, Table 6).

**Table 6:** Results of the two-way ANOVA for the survival percentage of adult caprellids after the 12 days experiment.

Source of variation	df	MS	F	*P*	F versus
Species (Sp)	1	34.72	0.15	0.0703	Res
Treatment (Tr)	2	1554.38	6.78	0.0107*	Res
Sp x Tr	2	345.72	1.51	0.2603	Res
Residual (Res)	12	229.11			
Cochran's test		C = 0.37 n.s.			
Transformation		None			
SNK		A = P; A≠D; P≠D			

*P<0.05.

A = Artemia; P = phytoplankton; D = detritus. See also Fig 5.

### Influence of diet on survival and growth rate of juveniles (Experiment 3)

**Fig 6:**
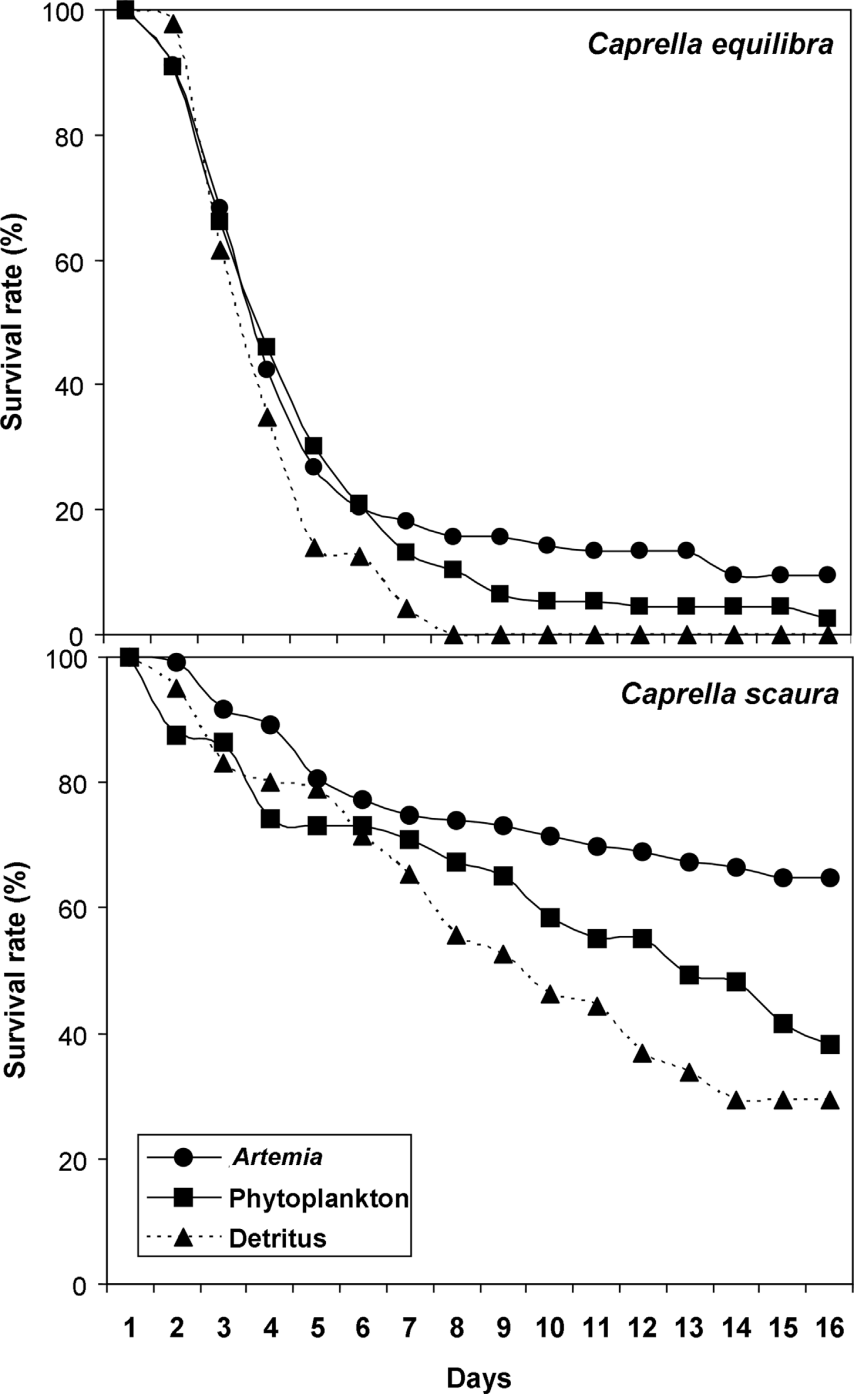
Survival rate (%) of juvenile specimens newly emerged from the brood pouch of *Caprella equilibra* and *C*. *scaura* after 16 days. Specimens were fed with *Artemia*, phytoplankton or detritus.

**Fig 7:**
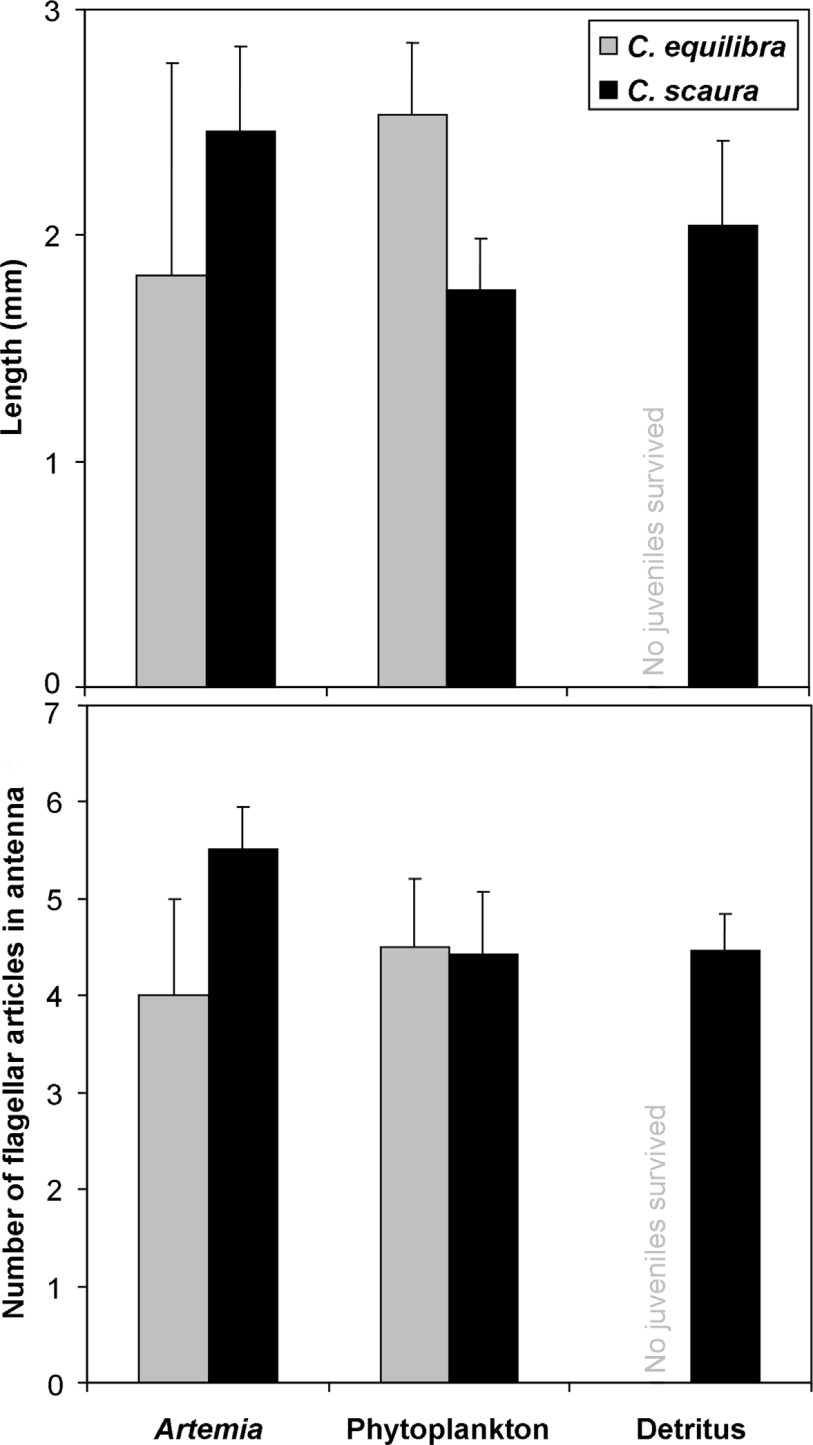
Body length (mm) and number of articles in the flagellum of antennae 1 of juveniles of *Caprella equilibra* and *C*. *scaura* after 16 days. Specimens were fed with *Artemia*, phytoplankton or detritus. Data for detritus in *C*. *scaura* is not provided since all the juveniles died before the end of the experiment.

In general terms, the invasive *Caprella scaura* showed higher values of survival than the native *C*. *equilibra* (Fig 6, Table 7). For both species, the highest values of survival were obtained for hatchlings fed with *Artemia* nauplii (64.7% and 9.4% respectively), followed by those fed with phytoplankton (38.2% and 2.4%) and detritus (29.5% and 0%). The two-way ANOVA showed significant differences between the *Artemia* treatment and the other two (Table 7). Despite the differences measured for survival rate, the juveniles which successfully reached the end of the experiment did not show any differences in body length among the three treatments (one-way ANOVA, *C*. *equilibra*: F = 0.17, p = 0.69, *C*. *scaura*: F = 1.95, p = 0.23) (Fig 7) with mean values ranging from 1.7 to 2.6 mm. Similarly, no significant differences among treatments were measured for the number of flagellar articles in antenna 1 (one-way ANOVA, *C*. *equilibra*: F = 0.13, p = 0.74, *C*. *scaura*: F = 3.99, p = 0.08) (Fig 7) with mean values ranging from 4.0 to 5.5. The number of flagellar articles in the antenna 1 was always two in newly born juveniles, plus three basal articles at the peduncle. Juveniles increased one article in the flagellum before each moulting, thus the number of articles indicated the number of moults, which ranged from 2 to 4 in both species. During the experiment, most of the *C*. *scaura* juveniles were attached to the female body showing parental care, while this behaviour was not observed for juveniles of *C*. *equilibra*.

**Table 7:** Results of the two-way ANOVA for the survival percentage of juvenile caprellids after the 16 days experiment.

Source of variation	df	MS	F	*P*	F versus
Species (Sp)	1	10193.63	32.66	0.0000***	Res
Treatment (Tr)	2	1685.63	5.40	0.01166*	Res
Sp x Tr	2	658.43	2.11	0.1432	Res
Residual (Res)	24	312.13			
Cochran's test		C = 0.34 n.s.			
Transformation		None			
SNK		A≠P; A≠D; P = D			

*P<0.05.

A = *Artemia*; P = phytoplankton; D = detritus. See also Fig 6.

## Discussion

### The nutritional value of caprellids: an adequate resource in aquaculture?

Although there is little information available regarding the relative nutritional value of caprellids for marine finfish, recent efforts have been directed towards resolving this. FA profiles have been characterised for several species [4,7,13,41,50,51] and a complete nutritional characterisation has been conducted for four common caprellids of the Strait of Gibraltar, including *C*. *equilibra* [7]. These authors proposed that the shallow water amphipods from the Strait of Gibraltar were suitable for use as natural live feed since their protein, lipid and carbohydrate contents are adequate for normal fish, crustacean and mollusc growth. They also pointed out that, in connection with lipid content, marine amphipods, especially caprellids, turned out to be more adequate for aquaculture than freshwater gammarids, based on their higher PUFA levels and polar lipids. Our results, based on the lipid analysis of *C*. *equilibra* and *C*. *scaura* from marinas, also show an adequate nutritional level.

When compared with data from existing literature for other live feeds, including enriched feeds, caprellids showed reasonably adequate nutritional profiles with regards to the lipid composition. Levels of PC or PE were similar to those measured for enriched rotifers [52] or copepods [53]. Levels of TAG were of the same order than those provided by copepods but lower than the levels measured for rotifers or *Artemia* [53]. Regarding the fatty acid composition, the levels of ARA and EPA in caprellids were higher than those measured in copepods, rotifers and *Artemia* [52,53]. Furthermore caprellids generally showed higher percentages of DHA than rotifers or *Artemia* [52,53], although demonstrated lower levels than copepods, which can reach 40% DHA [52].

Besides the use of caprellids as live feed, another feasible approach could involve the dehydration of caprellids for use as a component in the fabrication of fish feed or to be encased in microdiets. Diets for fish larvae are usually enriched with additional components thus in this sense, caprellids could potentially be used as a supplement of EPA and/or ARA. Overall, the suitability of caprellids as a feed resource for fin- and shellfish in aquaculture, based on an adequate nutritional composition, looks promising. However, we must also take into account that a high levels of specific fatty acids can also have detrimental effects. For example, ARA is an important component for diet [54], but ARA enriched live prey can induce albinism in Senegal sole (*Solea senegalensis*) larvae [55]. Additionally, further research into other important compounds for fish farming (such as amino-acids, selenium, etc) is necessary to properly address the suitability of caprellids as feed in aquaculture. The present study marks caprellids as organisms which at the very least should be compared in more detail to other traditionally used live feeds for suitability, but generally indicates that caprellids fed with detritus have an adequate lipid composition to be used as an alternative feed source in aquaculture.

### Detritus as feed for caprellid culture

The present study produces evidence supporting the use of detritus (composed mainly of fish faeces, and secondarily of uneaten feed pellets) for caprellid culture. In natural habitats, although amphipods form a trophic continuum from primary herbivores to carnivores [56], [27] and [16] found that detritus is the main feed item in the majority of species. The importance of detritus in benthic communities has often been reported in the literature, and is considered to be highly nutritious after a short period of microbial colonization [57]. Each of the three feed types used as a diet for feeding caprellids during the present study showed a distinct composition; detritus had higher levels of ST and 16:0, 18:0, OA, LA or DHA, while *Artemia* nauplii was richer in PC, TAG, 18:1(n-7) and EPA, and phytoplankton was a source of LNA and PC. The lower concentrations of lipid classes measured for phytoplankton could be due to the presence of other polar lipids such as glycolipids or betaine lipids [58], which were not quantified during the present study. High amounts of glycolipids in phytoplankton, and an abundance of free fatty acids in fish faecal faeces have also been measured (Hachero-Cruzado, unpublished data). In general terms, when compared with other diets described in literature for feeding amphipods or fishes (e.g. [13,42]), detritus can be considered a diet of relatively adequate nutritional value. It is a source of sterols (ST) 16:0, 18:0, and interestingly OA, LA and DHA. In fact, fragmented pellets and faeces might represent a new trophic source for fouling organisms, providing them with terrestrial fatty acids such as LA, which are unusual in marine environments [13]. These authors found that the sediments associated with fish farms presented higher percentages of OA and LA than control sediments, due to the faeces contribution, and suggested that faeces could be used as a trophic resource. Indeed, LA is considered a dietary essential fatty acid in the diets of vertebrates [42], since physiologically essential FAs, ARA (20:4n-6), EPA and DHA are obtained through diet or are synthesised from LA or LNA via a desaturase-elongase system [59]. [13] found that *Caprella equilibra* collected from fish farms were characterised by a higher concentration of PUFA(n-6) (mainly LA) than specimens from a control site. This data supports our results and the potential use of fish farm detritus as a resource. The FA profiles provided by [51] for *C*. *mutica* from aquaculture facilities (salmon sea cages, shellfish longlines stocked with mussels and mooring lines from caged finfish aquaculture) do not differ very much from those obtained during the present study for *C*. *equilibra* and *C*. *scaura* fed with detritus, with the exception of the higher concentrations of LA measured in our study.

It has been well established in recent decades that long chain PUFAs, especially (n-3) PUFAs, have a vital role in human nutrition, disease prevention and health promotion ([17,60] and references therein). They cannot be synthesised by fishes, meaning their levels depend upon dietary intake. Among these, the most important (n-3) PUFAs are EPA, DPA and DHA. These FAs cannot be produced by most crustacean species in sufficient quantities for metabolic functioning so they too must be obtained from the diet ([42] and references therein). The present study has shown that detritus seems to be an adequate source of DPA and DHA for caprellids, however a poor source of EPA. Detritus proved also to contain low levels of PC and TAG while *Artemia* was an excellent source of these lipids (see Table 2). Phospholipids, such as PC, located mainly in biological membranes, have an essential role in regulating biophysical properties, protein sorting and in cell signalling pathways [61,62]. The phospholipids of the majority of marine species including fish, molluscs and crustaceans are rich in PUFA, especially (n-3) [62]. The principal storage lipids in marine organisms including amphipods consist of TAGs, with a sufficient amount of these compounds ensuring the survival of individuals over the non-productive season [42]. The percentage of TAGs in relation to the amount of total lipids can show an increase in energy demand allocated to gamete production and egg incubation [63]. A relatively high amount of TAG can indicate periods of starvation, where storage lipids may be important for survival [64]. During the present study, adults fed with detritus had a higher survival rate than those fed with *Artemia* or phytoplankton, but interestingly the survival rate of hatchlings during the first 16 days of development showed the opposite pattern. The higher survival rate obtained for caprellid hatchlings fed with *Artemia* is probably related to higher requirements of TAG and/or PC during the first stage of life history, which cannot be provided by detritus or phytoplankton. A highest level of TAG in amphipod females, increasing with the maturation of the ovary, has been reported by [36] and it is expected that hatchlings also require higher TAG concentrations as a lipid reserve to properly face the first days of the life cycle. [36] also pointed out that MUFAs are used in egg development, and in the present study *Artemia* proves to be a good source of MUFA, especially 16:1, in comparison with detritus. Curiously, [41] found higher percentages of 16:1 in caprellid females than in males, so this fatty acid is probably related to egg development and it is required in higher levels by hatchlings. For all these reasons, a supply of *Artemia* would be optimal in completing the requirements of TAG, PC, and MUFA during the early stages of development of caprellid cultures. In field populations, these requirements are probably provided by small crustaceans such as copepods or other amphipods, such as those observed in the guts of specimens from the marinas examined during the present study. The results of [30] seem to support this hypothesis since these authors found significantly higher amounts of prey (copepods) in juveniles of *C*. *scaura* than in adults. A diet shift during the development has also been observed in other amphipods [65]. Ontogenetic shifts in diet may occur in order to overcome physiological constraints [66]. For instance, when juveniles have physiological limitation in the maximum rate of feed uptake, they might rely on higher quality sources of feed to minimise the amount of feed and maximise energy uptake [67]. Hence, the juveniles could satisfy their higher requirements of TAG, PC and MUFA with an increased copepod intake.

### Caprellid cultures and IMTA systems

The survival rate for adult specimens was higher for caprellids fed with detritus than for those fed with *Artemia* or phytoplankton. In the case of hatchlings, the results are not as promising due to higher mortalities seen in specimens subject to a diet of detritus. Despite this, hatchlings which successfully completed the juvenile stage with detritus were seen to reach similar sizes as those fed with *Artemia* or phytoplankton. Detritus therefore seems not to negatively affect the growing rate of survivors. For caprellids cultured under laboratory conditions at small- or medium-scales [25, 26] it is therefore recommended that a mixed diet of detritus enriched with *Artemia*, especially during the first 15 days of culture, is used.

If the culture is to be maintained offshore, the presence of a nearby supply of detritus would be recommended. Fish farms and sea-cage structures are interesting sources of detritus [68] so a caprellid culture associated to these aquaculture facilities could be an excellent option. In this case, the availability of detritus for caprellids is guaranteed, and the additional TAG, PC and MUFA requirements of juveniles and reproductive females could be obtained by preying on copepods or other small planktonic crustaceans, which naturally inhabit fish farm ecosystems. Copepods are among the dominant groups inhabiting the plankton communities of fish farms [69] and their biomass at net-cage areas can be higher when compared with control areas [70].

Copepods are frequently found in the gut contents of caprellids in field populations [27] and caprellids can prey directly on them, or ingest them accidentally while filtering (pers. obs.). Copepods are rich in lipids (e.g. [71]) and can be an important source of TAG [72]. In fact, cultured copepods have been successfully used in the larviculture of various marine fish larvae, although the upscale of copepod cultures to commercial levels is still a challenge [73].

The availability of detritus in fish farms, together with access to planktonic crustaceans naturally inhabiting the area, would probably provide an optimum diet. This would explain the reason that the highest population densities for caprellids have been recorded in substrates of aquaculture facilities (see [4] for details). On the west coast of Scotland, densities of 319 000 ind/m^2^ have been reported for *Caprella mutica* in a fish farm [74]. In Ireland and Scotland, *C*. *mutica* exhibits greater fecundity and abundance, size and population longevity at fish farm locations, with these results being attributed to organic enrichment from the fish farms [74,75,76]. The present study confirms experimentally that detritus in the form of fish faeces is an adequate feed, although also provides evidence that caprellids must have access to a source of TAG, PC and MUFA during the first stages of their life histories.

Encouraging the presence and abundance of bioremediating biota alongside net cage aquaculture has been proposed as a potential mechanism to ameliorate associated negative aquaculture impacts (see [4] and references therein). Although not for caprellids, estimates of filtration rate have been obtained for other amphipods. For example, the clearance rate of *Haploops nirae* (Kaim-Malka, 1976) has been estimated at ca. 15 mL h^-1^ ind ^-1^ [77]. The results from the present study justify further research into providing a quantitative estimation of how much detritus could be recycled by caprellids, since caprellids could be potential candidates for bioremediation.

In IMTA systems, the culture of caprellids alongside macroalgae could be advisable, since macroalgae provide substrate for caprellids. Caprellids may also have a direct benefit to macroalgae by reducing epiphytic overgrowth (e.g. [78,79]; see [4] for details). In addition to attaching to many different types of natural substrates [80], caprellids easily use a range of artificial ones such as buoys, nets, plastic mesh, ropes and PVC panels. [25] tested different kinds of plastic mesh with varying complexities for maintaining a culture of *C*. *scaura* and achieved very high densities (>10 000 ind/m^2^) in all the substrates. Therefore, the culture of caprellids in IMTA could only require the use of additional artificial structures, easy to set and remove, located close to the fish farms. Previous studies of water currents would be desirable to find the most adequate placement locations in order to maximise efficiency in receiving the detritus from the fish faeces and uneaten feed pellets.

The two caprellid species used in the present study, *C*. *equilibra* and *C*. *scaura* can reach very high densities when associated with fouling communities of marinas in Southern Spain [29, 32, 81]. Recently, *Caprella scaura* has also been found on off-coast fish farm cages in the Mediterranean Sea [82]. The life history of *Caprella equilibra* in small-scale laboratory conditions has been recently completed [40] and [4] considered *C*. *equilibra* as ideally suited to large-scale culture. A preliminary study of a medium-scale culture of *C*. *scaura* has also been conducted, showing promising results for this species [25]. In fact, during the present study hatchlings of *C*. *scaura* showed a significantly higher survival rate than those of *C*. *equilibra*. Consequently, *C*. *scaura* should also be a good candidate for large scale cultivation. However, it must be taken into account that *C*. *scaura* is a strongly invasive species which is quickly spreading across the Mediterranean and East Atlantic [34] and seems to be ecologically displacing the native *C*. *equilibra* [29] along European coasts. Therefore, as pointed out by [4] any research elucidating the aquaculture potential of caprellid species should adopt a “precautionary principle” assuming nonindigenous species may have detrimental impacts on indigenous biota (unless proven otherwise) and concentrate on indigenous and/or already established caprellid species, such as *C*. *equilibra*.

Provided that detritus is an adequate diet and that fish farms are an excellent source of detritus, the next step is to experimentally develop caprellid culture at a large-scale under controlled conditions in order to optimise and evaluate the use of detritus in terms of caprellid survival rates and reproductive success. Pilot experiments with caprellid cultures associated to IMTA systems are also needed to evaluate the biomass production and the economic and ecological sustainability of these cultures.
